# Monocular deprivation induces dendritic spine elimination in the developing mouse visual cortex

**DOI:** 10.1038/s41598-017-05337-6

**Published:** 2017-07-10

**Authors:** Yanmei Zhou, Baoling Lai, Wen-Biao Gan

**Affiliations:** 1 Drug Discovery Center, Key Laboratory of Chemical Genomics, Peking University Shenzhen Graduate School, Shenzhen, 518055 China; 2 Skirball Institute, Department of Neuroscience and Physiology, Neuroscience Institute, New York University School of Medicine, New York, NY 10016 USA

## Abstract

It is well established that visual deprivation has a profound impact on the responsiveness of neurons in the developing visual cortex. The effect of visual deprivation on synaptic connectivity remains unclear. Using transcranial two-photon microscopy, we examined the effect of visual deprivation and subsequent recovery on dendritic spine remodeling of layer 5 pyramidal neurons in the mouse primary visual cortex. We found that monocular deprivation (MD), but not binocular deprivation (BD), increased dendritic spine elimination over 3 days in the binocular region of 4-week-old adolescent mice. This MD-induced dendritic spine elimination persisted during subsequent 2–4 days of binocular recovery. Furthermore, we found that average dendritic spine sizes were decreased and increased following 3-day MD and BD, respectively. These spine size changes induced by MD or BD tended to be reversed during subsequent binocular recovery. Taken together, these findings reveal differential effects of MD and BD on synaptic connectivity of layer 5 pyramidal neurons and underscore the persistent impact of MD on synapse loss in the developing visual cortex.

## Introduction

Sensory experiences play an important role in the development and plasticity of the mammalian cortex^1–6^. Ocular dominance plasticity (ODP) in the visual cortex has been one of the best models for studying sensory-experience dependent plasticity^7^. During the critical period of visual cortex development, closing one eye (monocular deprivation: MD) for a few days causes rapid weakening of the responses to the deprived eye and subsequent strengthening of the responses to the non-deprived eye^8–10^. Binocular visual deprivation (BD) over days, however, has no substantial impact on neuronal responses^11, 12^. These studies suggest that competitive interactions between the inputs from the two eyes have a robust effect on functional responsiveness of neurons in the developing visual cortex^13^.

In addition to functional alterations, visual deprivation also induces structural changes of thalamocortical axon arbors^14–16^, intracortical horizontal connections^17, 18^ and postsynaptic dendritic spines^19–22^. It has been reported that BD for two weeks starting before the time of eye-opening has no effect on the density of protrusions of layer 5 pyramidal neurons, but increases spine motility at postnatal day 28^23^. During the critical period of the mouse visual cortex development (~postnatal day 25), MD for four days reduces the density of dendritic protrusions of layer 2/3 pyramidal neurons^24^. Furthermore, MD over days increases the formation of dendritic spines on apical tuft dendrites of layer 5 but not layer 2/3 pyramidal neurons in adult mouse visual cortex^25^. In addition, in the binocular region of the visual cortex of 3-week old rat, postsynaptic strength onto layer 2/3 pyramidal neurons decreases after 2 days of MD and increases after 6 days, but not 2 days, of BD^26^. Together, these studies indicate that MD or BD over days has significant effects on synaptic connectivity in the developing rodent visual cortex.

While studies so far have shown the effects of visual deprivation on the density of dendritic protrusions and synaptic strengths in the developing visual cortex, the precise roles of MD and BD in dendritic spine remodeling remain unclear. Specifically, it remains unknown whether and how MD and BD affect the processes of dendritic spine formation or elimination or both during the critical period of visual cortex development. Furthermore, although MD and BD have differential effects on neuronal responsiveness to visual stimuli during development, it is unclear whether the impacts of MD and BD on dendritic spine dynamics are similar or different. Moreover, when the initially closed eye is allowed to regain vision after MD, normal binocular response is restored in the developing visual cortex^27, 28^. Whether visual deprivation-induced synaptic changes could be reversed after subsequent binocular recovery remains unknown.

To better understand the impact of visual deprivation on synapse development and plasticity, we used transcranial two-photon microscopy to study the effects of MD and BD on postsynaptic dendritic spines of layer 5 pyramidal neurons in the visual cortex in transgenic mice expressing yellow fluorescent protein (YFP). Our studies reveal differential effects of MD and BD on synaptic connections and highlight long-lasting impact of MD on synapse elimination of layer 5 pyramidal neurons during the critical period of visual cortex development.

## Results

### Identify binocular and monocular regions of the mouse visual cortex

**Figure 1:**
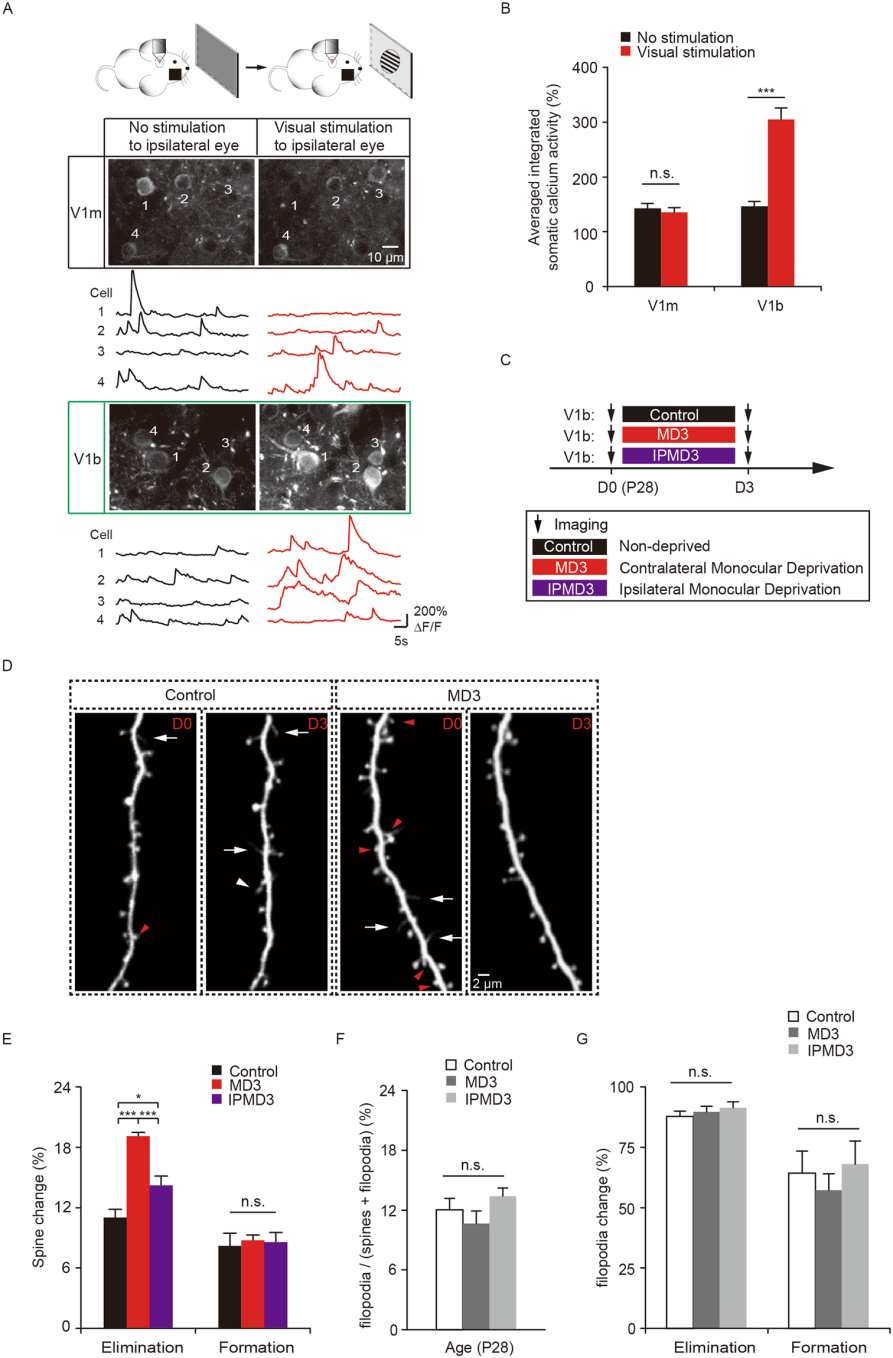
MD increases dendritic spine elimination in the binocular region during the critical period. (**A**) Two-photon calcium imaging of layer 2/3 pyramidal neurons in the monocular (V1m) and binocular (V1b) regions of the visual cortex in mice with the contralateral eye covered. Scale bar: 10 μm. Black traces represent activities without visual stimulation. Red traces represent activities when visual stimulus was presented to the ipsilateral eye. Calcium fluorescence traces of four neurons over 50 s are shown. (**B**) Somatic calcium activities in V1m were comparable between no stimulation and stimulation conditions (n = 4 mice, 205 cells). Somatic calcium activities in V1b were significantly increased when visual stimulus was presented to the ipsilateral eye (n = 3 mice, 179 cells). (**C**) Schematic of experimental paradigm. MD represents deprivation of the contralateral eye relative to the imaged hemisphere. IPMD represents deprivation of the ipsilateral eye. (**D**) Images of dendritic spines on apical tuft dendrites of layer 5 pyramidal neurons in control and MD mice over 3 days during the critical period. Red arrowheads indicate eliminated dendritic spines and white arrowhead indicates newly formed spines. White arrows indicate dendritic filopodia. Scale bar: 2 μm. (**E**) Percentage of dendritic spines eliminated and formed over 3 days in the binocular region. 3d of MD significantly increased spine elimination as compared to the non-deprived control and ipsilateral eye deprived mice, respectively (n = 1096 spines, 42 dendrites from 6 control mice, n = 4019 spines, 145 dendrites from 21 MD3 mice, n = 966 spines, 43 dendrites from 6 IPMD3 mice). In mice with the ipsilateral eye deprived for three days, significant spine elimination was observed as compared to the controls. No significant difference in spine formation among the three groups. (**F**) Percentage of filopodia over total dendritic protrusions (spines and filopodia) in the binocular region (n = 150 filopodia, 42 dendrites from 6 control mice; n = 384 filopodia, 106 dendrites from 16 MD3 mice; n = 149 filopodia, 42 dendrites from 6 IPMD3 mice). (**G**) Percentage of filopodia eliminated and formed over 3 days in the binocular region. Data are presented as mean ± SEM. *P < 0.05, ***P < 0.001, n.s. = not significant.

To study how visual deprivation affects synaptic structural plasticity, we first used two-photon Ca^2+^ imaging to confirm the monocular and binocular regions of the mouse primary visual cortex based on the stereotaxic coordinates of the mouse brain^29, 30^. In this experiment, awake head-retrained mice were exposed to visual stimuli with the contralateral eye covered under a two-photon microscope (Fig. 1A). We then imaged the activities of somata of layer 2/3 pyramidal neurons expressing the genetically encoded calcium indicator GCaMP6s. Previous studies have shown that neurons in the monocular region receive inputs only from the contralateral eye, whereas neurons in binocular region receive inputs from both eyes^11, 29^. Consistent with these studies, when visual stimuli were presented to the ipsilateral eye, we found that neurons in the monocular region did not exhibit significant increases in somatic activities when compared to the non-stimulated condition (P = 0.11, Fig. 1A and B). On the other hand, somatic activities of neurons in the binocular region were significantly increased upon visual stimulation to the ipsilateral eye (P < 0.001, Fig. 1A and B). These results confirm that the areas we imaged based on the stereotaxic coordinates of the mouse brain corresponded to the monocular and binocular regions of the primary visual cortex.

### MD increases dendritic spine elimination in the binocular region during the critical period

To investigate how visual deprivation affects synaptic remodeling in the developing visual cortex, we used transcranial two-photon microscopy to image postsynaptic dendritic spines on the apical dendrites of layer 5 pyramidal neurons over time in the binocular region of 4-week-old transgenic mice expressing YFP (Fig. 1C and D). Monocular deprivation via eyelid closure of the contralateral eye at the peak of the critical period (postnatal day 28) for three days significantly increased dendritic spine elimination in the binocular region, as compared to that in non-deprived control mice (19.1 ± 0.4% versus control 11.0 ± 0.8%, P < 0.001). In contrast, spine formation rate was not significantly altered by MD during the same period (8.7 ± 0.5% versus control 8.2 ± 1.2%, P = 0.45) (Fig. 1E). Furthermore, when the ipsilateral eye was deprived for three days, a significant increase in spine elimination, but not formation, was also observed when compared to that in non-deprived controls (elimination: 14.2 ± 0.9%, P < 0.05; formation: 8.6 ± 1.0%, P = 0.75) (Fig. 1E). Spine elimination induced by ipsilateral-eye MD was significantly lower than that caused by contralateral-eye MD (P < 0.001) (Fig. 1E). Together, these results reveal the impact of MD on the pruning of synaptic connections of layer 5 pyramidal neurons in the binocular region during the critical period of development.

In the developing cortex, dendrites contain dendritic spines as well as filopodia, which are long and thin protrusions without a bulbous head^31–33^ (Fig. 1D). Consistent with previous study^31^, we found that ~10–13% of dendritic protrusions were filopodia in the visual cortex of 4-week-old mice (control 12.0% ± 1.2% versus MD3 mice 10.7% ± 1.2% and IPMD3 mice 13.4% ± 0.8%, P = 0.16) (Fig. 1F). MD for three days had no significant effect on filopodia elimination (control 87.9% ± 2.1% versus MD3 89.6% ± 2.5% and IPMD3 91.4% ± 2.4%, P = 0.74) or formation (control 64.4% ± 9.0% versus MD3 57.2% ± 6.8% and IPMD3 68.1% ± 9.4%, P = 0.50) (Fig. 1G). These results indicate that unlike dendritic spines, monocular deprivation over 3 days has no significant effect on the dynamics of dendritic filopodia.

### MD-induced spine elimination depends on the competitive interactions between the deprived and non-deprived eye

**Figure 2:**
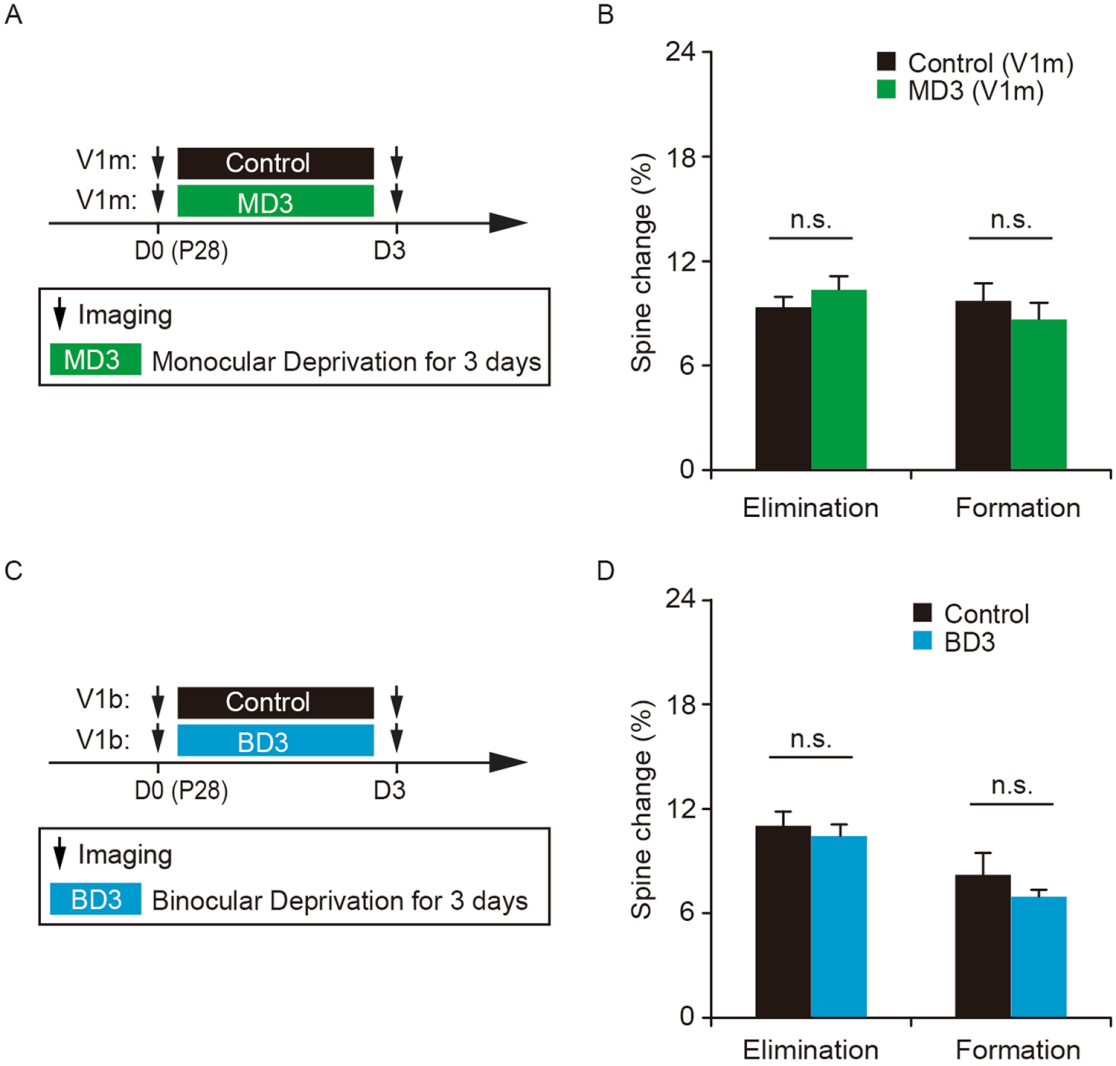
MD-induced spine elimination depends on the competitive interactions between the deprived and non-deprived eye. (**A**) Schematic of experimental design to examine the effect of MD in the monocular region of the primary visual cortex. (**B**) No significant difference in spine elimination or formation over 3 days between MD mice and non-deprived control mice (n = 713 spines, 30 dendrites from 4 MD mice, n = 653 spines, 30 dendrites from 4 control mice). (**C**) Schematic of experimental design to examine the effect of BD in the binocular region of the primary visual cortex. BD represents binocular deprivation. (**D**) No significant difference in spine elimination or formation over 3 days between BD and non-deprived control mice (n = 1023 spines, 47 dendrites from 6 BD mice, n = 1096 spines, 42 dendrites from 6 control mice). Data are presented as mean ± SEM. n.s. = not significant.

To better understand the effect of MD, dendritic spine turnover was examined after MD for 3 days in the monocular region of the primary visual cortex (V1M) in 4-week-old mice (Fig. 2A). We found that 3-day monocular deprivation of the contralateral eye did not cause significant changes in spine elimination or formation in the monocular region (elimination: 10.4 ± 0.8% versus control 9.3 ± 0.6%, P = 0.25; formation: 8.6 ± 1.0% versus control 9.7 ± 1.0%, P = 0.39) (Fig. 2B). Thus, the effect of MD on spine elimination is limited to the binocular zone of the primary visual cortex, suggesting that MD-induced spine elimination is not simply due to the lack of visual inputs from the deprived eye, but instead depends on the competitive interactions between the inputs from the deprived and non-deprived eye.

To further investigate the effect of the competitive interactions between the two eyes’ inputs, we examined the effect of BD on dendritic spine dynamics in the binocular region of 4-week old mice (Fig. 2C). Unlike MD for 3 days, BD for 3 days had no significant effect on the degree of spine elimination or formation as compared to non-deprived controls (elimination: 10.4 ± 0.7% versus control 11.0 ± 0.8%, P = 0.47; formation: 6.9 ± 0.4% versus control 8.2 ± 1.2%, P = 0.63) (Fig. 2D). This lack of BD effect on spine elimination further suggests that the competition between the deprived and non-deprived eye inputs is critical for synaptic structural plasticity.

### MD-induced spine elimination persists during subsequent binocular recovery

**Figure 3:**
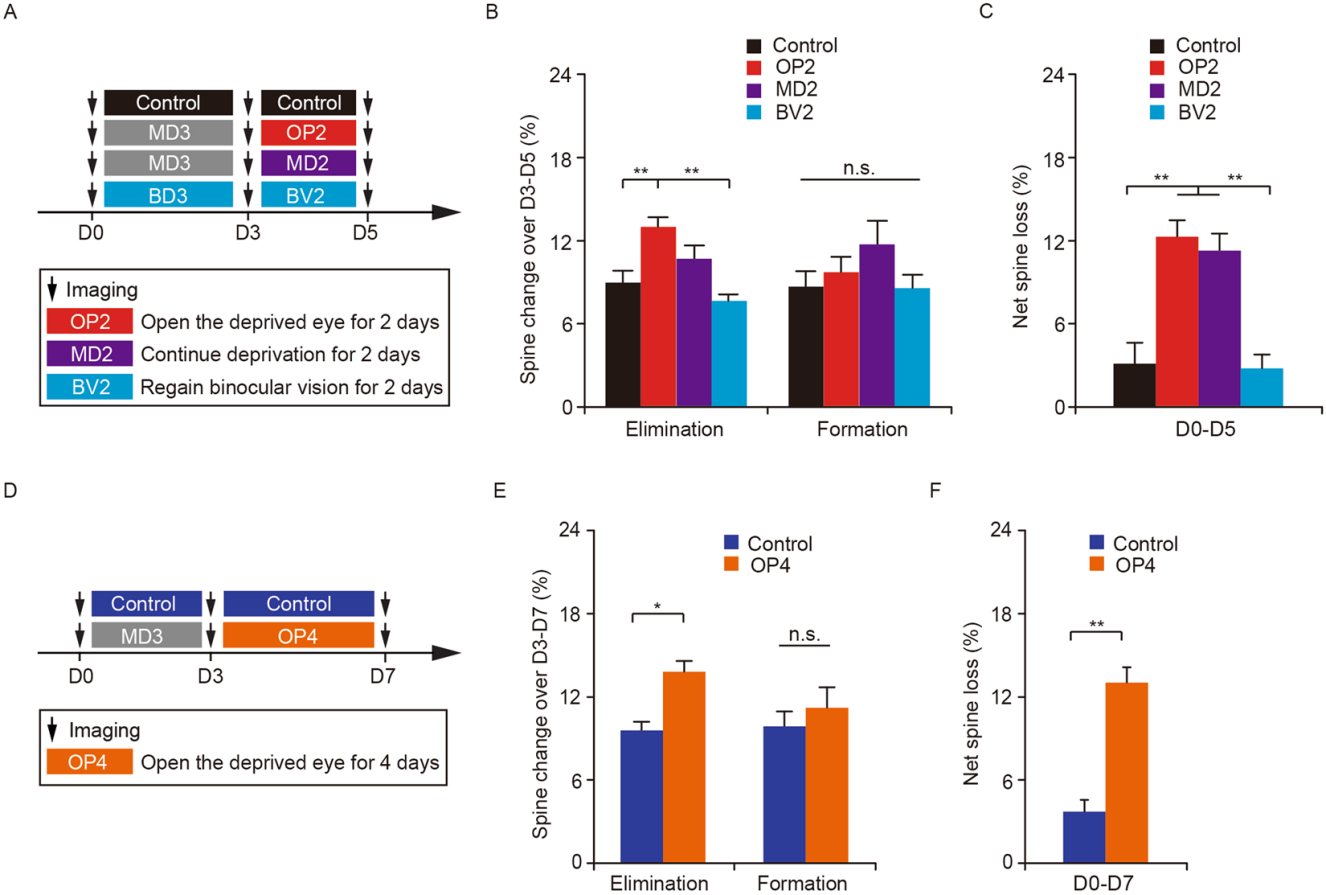
MD-induced spine elimination persists during subsequent binocular recovery. (**A**) Schematic of experimental paradigm. OP2 represents the condition in which the contralateral deprived eye was reopened for two days. MD2 represents continuous deprivation of the contralateral eye for two days and BV2 represents regaining binocular vision from BD for two days. (**B**) Percentage of dendritic spines eliminated and formed between days 3 and 5. The rate of spine elimination in OP2 mice (n = 1539 spines, 58 dendrites from 9 mice) was comparable to that in MD2 mice (n = 1349 spines, 58 dendrites from 8 mice), but significantly higher than that in non-deprived control mice (n = 1067 spines, 42 dendrites from 6 mice) and BV2 mice (n = 839 spines, 38 dendrites from 5 mice). No significant difference in the rate of spine formation was observed among the four groups. (**C**) Changes in spine number over five days under various conditions. The degree of net spine loss in OP2 mice (n = 9) was comparable to that in MD2 mice (n = 7), but significantly higher than that in non-deprived mice (n = 6) and BV2 mice (n = 5). (**D**) Schematic of experimental design. OP4 represents the condition in which the contralateral deprived eye was reopened for four days. (**E**) Percentage of dendritic spines eliminated and formed between days 3 and 7. The rate of spine elimination in OP4 mice (n = 871 spines, 34 dendrites from 5 mice) was significantly higher than that in non-deprived control mice (n = 964 spines, 58 dendrites from 6 mice). There was no significant difference in spine formation between OP4 and control mice. (**F**) The degree of net spine loss in OP4 mice (n = 5) was significantly higher than that in non-deprived mice (n = 6). Data are presented as mean ± SEM. *P < 0.05, **P < 0.01, n.s. = not significant.

In the developing visual cortex, restoring binocular vision after monocular deprivation leads to the recovery of neuronal responses^22, 34^. To examine whether MD-induced spine elimination might be reversed upon binocular recovery, we reopened the deprived eye and imaged the same apical dendrites of layer 5 pyramidal neurons over a 2-day period of binocular recovery (Fig. 3A). We found that after the contralateral deprived eye was reopened for two days (OP2), the rate of spine elimination was comparable to that in mice with continued MD (MD2) (13.0 ± 0.7% versus MD2 mice 10.7 ± 1.0%, P = 0.08), and was significantly higher than that in age-matched non-deprived control mice (9.0 ± 0.9%, P < 0.01) or mice regaining binocular vision from BD (BV2) (7.6 ± 0.5%, P < 0.01) (Fig. 3B). There was no significant difference in the rate of spine formation among the four groups (OP2 mice 9.7 ± 1.1% versus controls 8.7 ± 1.1%, P = 0.56; MD2 mice 11.7 ± 1.7%, P = 0.39; BV2 mice 8.6 ± 1.0%, P = 0.55) (Fig. 3B). Over five days, a net spine loss in mice with binocular recovery from MD was comparable to that in continuously deprived mice (12.3 ± 1.2% versus MD2 mice 11.3 ± 1.2%, P = 0.49), but was significantly higher than that in non-deprived control mice (3.1 ± 1.5%, P < 0.01) and mice regaining binocular vision from BD (2.8 ± 1.0%, P < 0.01) (Fig. 3C). These results suggest that the spine loss caused by 3-day MD does not return to the pre-MD level during subsequent two-day visual recovery.

To determine whether MD-induced spine elimination could be reversed after longer period of binocular recovery, we reopened the deprived eye for 4 days (Fig. 3D). We found that during the 4-day binocular recovery period, the degree of spine elimination was still significantly higher than that in age-matched non-deprived mice (13.8 ± 0.8% versus control 9.6 ± 0.6%, P < 0.05) (Fig. 3E). In contrast, the degree of spine formation was comparable to that in control mice (11.2 ± 1.5% versus control 9.9 ± 1.1%, P = 0.46) (Fig. 3E). Over seven days, a net spine loss in mice with binocular recovery from MD was also significantly higher than that in control mice (13.0 ± 1.1% versus control 3.7 ± 0.9%, P < 0.01) (Fig. 3F). Together, these results indicate that during the critical period of visual cortex development, the effect of MD on spine elimination of dendritic spines of layer 5 pyramidal neurons is persistent.

### MD or BD-induced spine size changes tend to be reversed after binocular recovery

**Figure 4:**
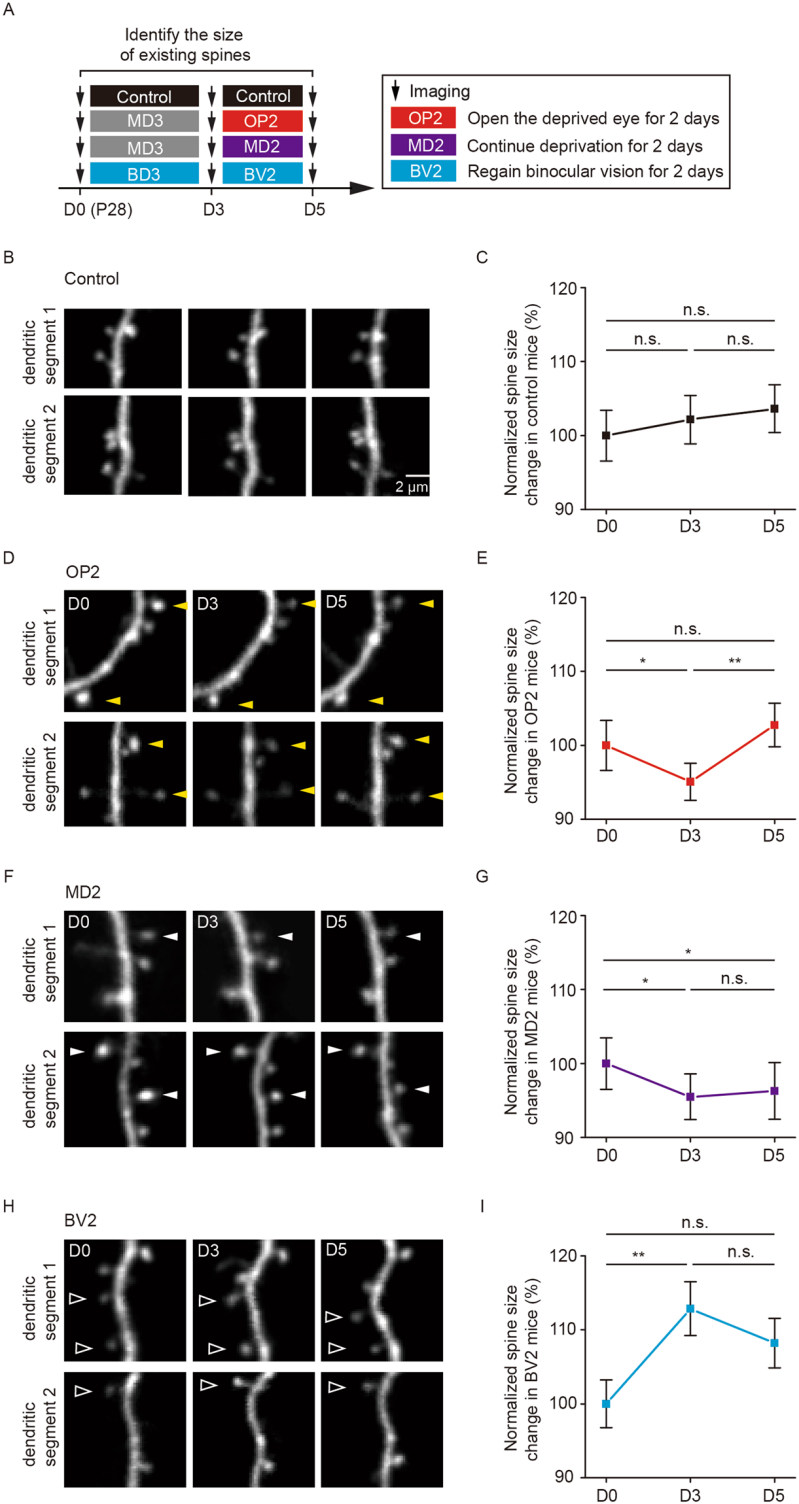
MD or BD-induced spine size changes tend to be reversed after binocular recovery. (**A**) Schematic of experimental design to evaluate the effect of visual deprivation and subsequent recovery on the size of dendritic spines in the binocular region of the primary visual cortex. (**B**) Images of dendritic spine size changes from day 0 to 5 in non-deprived control mice. Scale bar: 2 μm﻿. ﻿(**C**) Average spine size (measured by spine fluorescence relative to shaft fluorescence) exhibited no significant difference over 5 days in control mice (n = 116 spines from 6 mice). (**D**) Images of dendritic spine size changes from day 0 to 5 in OP2 mice. Yellow arrowheads indicate spines with size decrease after MD and size increase after subsequent binocular recovery. (**E**) Average spine size decreased during 3 days of MD and increased after 2 days of binocular recovery in OP2 mice (n = 145 spines from 5 mice). (**F**) Images of dendritic spine size changes from day 0 to 5 in MD2 mice. White arrowheads indicate spines with size decrease after MD and subsequent binocular recovery. (**G**) Average spine size showed no increase in mice with continued MD for additional 2 days (n = 125 spines from 5 mice). (**H**) Images of dendritic spine size changes from day 0 to 5 in BV2 mice. Open arrowheads indicate spines with size increase after BD over 3 days and decrease after subsequent binocular recovery. (**I**) Average spine size increased after 3 days of BD and showed a tendency to decrease after 2 days of binocular recovery in BV2 mice. Average spine size was not significantly different between day 0 and day 5 in BV2 mice. (n = 107 spines from 4 mice). Data are presented as mean ± SEM. *P < 0.05, **P < 0.01, n.s. = not significant.

In addition to investigating the effect of visual deprivation on synaptic remodeling, we also examined the size of individual spines over the periods of MD or BD and subsequent binocular recovery (Fig. 4A). No significant changes in spine size were observed in the binocular region of non-deprived control mice over 3 days or 5 days (P = 0.67 over the first three-day period, P = 0.55 over 5 days) (Fig. 4B and C). Notably, we found that average dendritic spine size underwent a significant decrease after 3d of MD (P < 0.05) and returned to the pre-deprivation level after a two-day period of binocular recovery (P < 0.01) (Fig. 4D and E). In contrast, mice with continued MD did not show significant changes in average spine size over the next two days (P = 0.64) and exhibited a significant drop in spine size over a total deprivation period of 5 days (P < 0.05) (Fig. 4F and G). In addition, we found that 3d of BD resulted in a significant increase in spine size in the binocular region (P < 0.01) (Fig. 4H and I). Following two days of binocular vision, the average spine size tended to be reduced and was comparable to that before BD (P = 0.07) (Fig. 4H and I). Because the dendritic spine size is strongly correlated with synapse strength^25, 35^, these findings suggest that 3-day MD reduces synaptic strength, while 3-day BD increases synaptic strength of layer 5 pyramidal neurons in the developing visual cortex. Unlike persistent spine elimination induced by MD, the changes in synaptic strength occurring during MD or BD tend to be reversed following a brief period of binocular recovery.

## Discussion

In this study, we show that during the critical period of the mouse visual cortex development, 3d of MD increases dendritic spine elimination and decreases spine size of layer 5 pyramidal neurons in the binocular region. In contrast, 3d of BD has no significant effect on spine elimination or formation, but significantly increases spine size. Restoring binocular vision tends to reverse spine size changes induced by MD or BD, whereas MD-induced spine elimination persists. These findings reveal differential effects of MD and BD on synaptic connectivity of layer 5 pyramidal neurons and underscore long-lasting impact of MD on synaptic connections during the visual cortex development.

Despite the potent effect of MD on neuronal responsiveness during the critical period of visual cortex development, the effect of MD on synaptic connections of pyramidal neurons remains poorly understood. Previous study has shown that MD from postnatal day 25–26 reduces the density of dendritic protrusions (spines and filopodia) of layer 2/3 pyramidal neurons in the mouse visual cortex^24^. But it is unclear whether such a reduction in dendritic protrusion density is due to increased dendritic protrusion elimination or reduced formation or both. Our studies show that MD over 3 days significantly increases dendritic spine elimination without affecting spine formation of layer 5 pyramidal neurons in the binocular region of 4-week-old mice, resulting in a net synapse loss of pyramidal neurons during the critical period of development. Furthermore, MD for three days has no significant effect on the turnover of dendritic filopodia, indicating that filopodia and spines of layer 5 pyramidal neurons have different susceptibilities to manipulations of visual experience during development. In addition to spine elimination, we also find that MD causes a significant reduction of dendritic spine size of layer 5 pyramidal neurons in the binocular region. Because 2–3 days of MD primarily causes a large reduction of the response to the deprived eye, resulting in a shift in ocular dominance of layer 5 pyramidal neurons^11^, our studies suggest that MD-induced spine elimination and size reduction may be functionally related to the deprived eye and likely contribute to the reduction of deprived eye responses.

In contrast to the effect of MD, our studies show that BD for three days has no significant effect on spine elimination or formation, but causes a significant increase in spine size of layer 5 pyramidal neurons during the critical period of visual cortex development. These findings are consistent with previous studies on the differential effects of MD and BD over days on neuronal responses^11, 12^. Because the dendritic spine size strongly correlates with synapse strength^36, 37^, our finding indicates that BD over 3 days leads to potentiation of synaptic connections of layer 5 pyramidal neurons. Previous study has shown that BD between 3–6 days results in a modest increase in synaptic strength of layer 2/3 pyramidal neurons in the binocular region of developing rat visual cortex^26^. Furthermore, BD over 5 days leads to increased cell responsiveness of layer 2/3 pyramidal neurons to both eyes in the binocular region of developing mouse visual cortex^30^. Thus, BD over 3 days or longer appears to cause a general increase in synaptic strength of pyramidal neurons in the developing visual cortex.

Our results show that MD increases dendritic spine elimination of layer 5 pyramidal neurons in the binocular region of the visual cortex during the critical period of development. Recent study has shown that MD over 4 days has no effect on spine gain or loss but increases the loss of inhibitory spine synapses of layer 2/3 pyramidal neurons in the binocular region of the adult visual cortex^21^. These findings raise the possibility that MD or BD may have differential impacts on the dynamics of inhibitory and excitatory synapses on layer 5 pyramidal neurons during development and/or adulthood. Further studies are needed to address this issue to better understand the role of sensory deprivation in modulating neuronal connectivity and function in the visual cortex.

In the developing visual cortex, restoring binocular vision by reopening the initially closed eye induces functional recovery from an ocular dominance shift^27, 38^. We find that following binocular recovery, the reduction of spine size caused by MD is reversed and returns to the pre-MD level. Furthermore, after regaining binocular vision from BD, the increased spine size induced by BD tends to return to the pre-deprivation level. This reversal of synapse strength may contribute to the functional recovery of deprived eye after MD and BD. Notably, our observations show that the reduction in dendritic spine number of layer 5 pyramidal neurons induced by 3d of MD does not return to the pre-MD level during subsequent 2–4 days of binocular recovery. This finding suggests that MD for several days could cause long-lasting changes in synaptic connections on apical dendrites of layer 5 pyramidal neurons in the developing visual cortex. The functional impact of this persistent spine elimination after MD remains unclear. Previous study has shown that MD during either the critical period or adulthood facilitates ocular dominance plasticity induced by MD later in adult life^27^. The persistent spine elimination induced by MD in our study may contribute to this prior experience-dependent enhancement of cortical plasticity induced by adult MD. Future studies to determine whether spines eliminated after MD are functionally related to the deprived eye would help to determine the long-term impact of MD-induced spine elimination on cortical circuit function.

Many lines of evidence suggest that the effect of MD is due to competitive interactions between inputs from the deprived eye and non-deprived eye, rather than overall lack of visual drive^10, 24, 39^. The reduced response of the deprived eye is thought to be caused by Hebbian-dependent weakening of the deprived eye during the first few days of MD^6, 7, 13, 40–43^. Consistent with this view, our findings show that 3d of MD, but not BD, increases spine elimination and reduces spine size in the binocular region, where inputs from the two eyes interact. Furthermore, in the monocular region that receives inputs exclusively from the deprived eye, 3d of MD has no significant effect on spine elimination or formation in 4-week-old mice. In contrast to the effect of MD, 3d of BD has no effect on spine elimination but leads to a significant increase in spine strength of layer 5 pyramidal neurons. This BD-induced increase in synaptic strength is thought to be due to homeostatic mechanisms that facilitates synapse strengthening to compensate for the loss of visual drive^26, 30, 44^. Further mechanistic studies are needed to determine how MD and BD lead to dendritic spine elimination, weakening or strengthening in order to better understand the fundamental role of sensory experience in the visual cortex development.

## Materials and Methods

### Experimental animals

Three-week-old C57BL/6 mice for calcium imaging were purchased from Guangdong medical laboratory animal center. Transgenic mice expressing Yellow Fluorescent Protein in layer 5 pyramidal neurons (YFP-H-line) were purchased from the Jackson Laboratory and housed in the animal facility of Peking University Shenzhen Graduate School (PKUSZ). Young (postnatal day 27–28) YFP-H-line mice were used in this study. All experiments were approved by and performed in accordance with the guidelines of the Animal Care and Use Committee at Peking University Shenzhen Graduate School.

### Two-photon calcium imaging

Two-photon calcium imaging of layer 2/3 somata in the primary visual cortex was carried out in awake, head-restrained mice. Genetically-encoded calcium indicator GCaMP6s was used to identify the binocular and monocular regions of the mouse visual cortex. A total amount of 0.1–0.2 μl of AAV-synapsin-GCaMP6s (AAV 2/1; >2 × 10^13^ (GC/ml) titer; from the University of Pennsylvania Gene Therapy Program Vector Core) was diluted two times in ACSF and slowly injected (Picospritzer III; 20 p.s.i., 20 ms, 0.3 Hz) over 10–15 min into layer 2/3 (depth of 200–300 μm) of visual cortex (2.6–3.5 mm posterior from bregma and 2.6–3.5 mm lateral from midline) using a glass microelectrode. 17 days after virus injection, mice were prepared for calcium imaging.

Surgical preparations for imaging were performed according to previously published studies^45–47^. Briefly, mice were anesthetized with pentobarbital sodium (80 mg/kg, i.p.). The mouse head was shaved and the skull surface was exposed, cleaned, glued and attached with two light metal bar head holder. Except the region near the identified visual cortex, the rest of skull and holder were sealed with dental acrylic cement. The exposed visual cortex skull was protected with 1% agarose when the dental acrylic cement was dry. Calcium imaging was performed 24 hours after surgery recovery.

To image somata at the depth of ~250 μm below the pial surface, the skull overlying the visual cortex of interest (2 mm × 2 mm) was removed and replaced with a glass window right before two-photon imaging (Olympus Fluoview 1000 two-photon system equipped with MaiTai DeepSee Ti:Sapphire laser from Spectra Physics). Round-shaped and square-wave black/white drifting gratings (0.08 cycles per degree, 4 cycles per second, covering 32° × 32° screen area as seen by the mouse) of changing orientations (8 directions) were used as visual stimuli. The stimuli were only presented to the ipsilateral eye in front of the central visual field of the mouse with the contralateral eye covered by a shade cloth (75 Hz refresh rate of the monitor, 18 cm away from the mouse’s ipsilateral eye). Mice were subjected to non-stimulation and visual stimulation environment two times (1 min/time), while calcium imaging was performed simultaneously. For GCaMP6 imaging, the laser was tuned to the wavelength of 920 nm with laser power of ~20 mW on the tissue sample. Calcium signals were recorded at 2 Hz using a 25× objective (N.A. 1.05, 2X digital zoom).

### Surgery for two-photon imaging of dendritic spines and visual deprivation

The surgical procedures for two-photon imaging of dendritic spines were performed according to previous published studies^45–47^. Before imaging, mice were deeply anesthetized with an intraperitoneal injection of pentobarbital sodium (80 mg/kg). The mouse head was shaved and the skull surface was exposed with a midline scalp incision. The periosteum tissue over the skull surface was removed without damaging the temporal and occipital muscles. The imaged binocular region (3.0 mm posterior to bregma, 3.0 mm lateral from midline) and monocular region (3.1 mm posterior to bregma, 2.6 mm lateral from midline) were identified based on stereotactic coordinates and marked with a fine marker. A thin layer of cyanoacrylate-based glue was first applied around the center of a head holder. The head holder was then immediately mounted to the top of the marked skull with the marked region exposed. After surgery, a high-speed micro-drill and microsurgical blade were used to thin a circular area over the marked region to a thickness of approximately 20 micrometers. The thinning procedure generally took less than 2 minutes. The skull was immersed in artificial cerebrospinal fluid (ACSF) and the head-restrained animal was then placed on the stage of a two-photon laser scanning microscopy.

For MD or BD, the right eyelid (contralateral to the imaged hemisphere) or both eyelids was sutured shut immediately after the first imaging session. Suture was checked each day and if not intact, animals were discarded. The eye was reopened three days later, immediately following the second imaging session. Mice were kept warm with a heating pad until recovered from anesthesia and then released to its original cage.

### Two-photon spine imaging

Apical tuft dendrites of layer 5 pyramidal neurons were reimaged over a period of 3–5 days. Image stacks of dendritic segments located in the superficial cortical layer were obtained using an Olympus two-photon microscope (FV1000MPE) with the laser tuned to 920 nm and with a 25X objective (N.A. 1.05) immersed in ACSF. A 3X digital zoom was used to yield high-magnification images (169 μm × 169 μm; 1024 pixel × 1024 pixel, 0.75 μm Z-step size) suitable for quantification of dendritic spines. For multiple imaging, the above procedure was repeated and the localization of the same region was facilitated by low-magnification images stacks at 1X digital zoom (508.43 μm × 508.43 μm; 1024 pixel × 1024 pixel, 2 μm Z-step size) and with reference to vascular landmarks under the thinned skull area. The animal was head restrained during image acquisition, which lasted ~30 minutes. The scalp incision was immediately sutured after imaging session.

### Data analysis

#### Calcium imaging

Neuronal calcium activity, indicated by GCaMP6 fluorescence changes, was analyzed post hoc using ImageJ software (NIH). The GCaMP6 fluorescence (F) during no stimulation and visual stimulation conditions was measured by averaging pixels within each visually identifiable soma (Regions of interests, ROIs). Changes of fluorescence ∆F/F_0_ was calculated as ∆F/F_0_ = (F − F_0_)/F_0_, in which ∆F was F − F_0_ (all F values were subtracted from a background fluorescence) and F_0_ was the average of 10% minimum F values over 1 min period, representing baseline fluorescence. ∆F/F_0_ above three times the standard deviation (SD) of baseline fluorescence was summed to measure somatic calcium activity of layer 2/3 neurons.

#### Spine structural plasticity

The procedure for quantifying spine dynamics has been described in the earlier studies^31, 45, 46^. Briefly, image stacks were analyzed using NIH Image J software. For each dendritic segment analyzed, filopodia were identified as long, thin protrusions with ratio of head diameter to neck diameter <1.2:1 and ratio of length to neck diameter >3:1. The remaining protrusions were classified as spines. Spines were considered the same between views if their positions remained the same distance from relative adjacent landmarks. Spines were considered different if they were more than 0.7 μm away from their expected positions based on the first view. More than 150 spines were analyzed from each animal. The minimum length of dendritic branches included in the analysis was over 30 μm. The degree of spine formation or elimination was calculated as the number of spines added or eliminated divided by the number of pre-existing spines.

Spine head size was measured according to previous studies^25, 48–50^. To correct for varying imaging conditions, the ratio of spine head diameter to adjacent dendritic shaft diameter was used to measure spine head diameter in tuft dendrites expressing YFP. After background subtraction, the fluorescence intensity of the spine (the intensity of all pixels covering the spine in the best focal plane) was divided by the fluorescence intensity of the adjacent dendritic shaft. Specifically, fluorescence intensity of a spine was measured as follows, where Area was the number of pixels in an oval surrounding the head of the spine and mean optical density (Mean OD) was the mean brightness of pixels in that area:

The ratio of spine head diameter to adjacent dendritic shaft diameter = (Area (of spine) × Mean OD (of spine) − Area (of spine) × Mean OD (of background))/(Area (of spine) × Mean OD (of dendrite) − Area (of spine) × Mean OD (of background)). The Mean OD of both the background and the dendrite was calculated from measurements taken next to each spine, averaged for each dendrite segment.

Spine size change was calculated by comparing spine size measurement between imaging sessions.

#### Statistics

All imaging data were presented as mean ± s.e.m. Tests for differences between groups were performed using non-parametric tests (two-tailed). Wilcoxon–Mann–Whitney test was used in Fig. 1B and E and Fig. 2B and D and Fig. 3B,C,E and F. In Fig. 1F and G, Kruskal-Wallis H test was used. In Fig. 4C,E,G and I, Wilcoxon test (2 related samples) was used. Significant levels were set at *P* ≤ 0.05. All statistical analysis was performed using the SPSS.
